# Letter Concerning the Southern Dental Association

**Published:** 1877-11

**Authors:** 


*Editor American Journal of Dental Science.*

DEAR SIR.—*A Correction.*—I see a letter published in your valuable journal, over the signature of H. that does some injustice to the Southern Dental Association.

He says "for the last two or three years, no quorum has been in attendance, and that the annual meeting that was to have been held at Montgomery, Ala., in May last, was adjourned at the instance of the executive committee."

Why H. should have made the above statement is marvelous indeed. Can it be that he has been made the dupe of some one, or did he say it on his own responsibility without knowing anything about the matter? The latter is hard to believe. The last three annual meetings have been small, 'tis true, as I can testify, having been present at all of them, but we had a quorum at each and more, and the usual proceedings were gone through with, and with much apparent interest to all present. The meeting at Montgomery, was a very interesting one as all present will say. The first day was rather dull, owing to the heavy rain fall the night before, preventing some getting in on time, but to say "no quorum" and "adjourned by the executive committee" is wonderful. A huge committee to elect officers, and change the time of meeting to July, 1878, and fix Atlanta, Ga. as the place, and still more, call a special meeting at Deer Park. H. must think we have very efficient executive committees down this way to do all that.

His reasoning as to the "falling off of membership," is also incorrect. It is not due to the "growing poverty of the South," for this is not true; the South is getting better off every year, and as a people, are better off to-day than they have been since the war. The true cause in my judgment is, that when this organization failed to grind satisfactorily for some who were rather aspiring in their composition, said aspirants had no further use for the organization, and it has been abandoned by them to drag out a feeble existence, as a child from parents whose blood is not pure. Be this true or not, I must say I hail with delight the National Convention now proposed by so many prominent men of the profession, of purer blood and nobler aspirations, who can esteem others equal to, if no better than themselves. L.

[The information as to the status of the Southern Dental Association was furnished our correspondent, so he informs us, by parties deemed reliable. and was of such a character as any journalist would feel justified in accepting as conclusive. We are pleased to hear from " L," that the "Southern," which has our heartiest sympathies and good wishes, is not dead or dying. As to the falling off in attendance upon its annual meetings, we cannot judge, not knowing, but it may be that both our esteemed correspondents are right in part. We hope that the aspirants for fame will not allow their desires in that direction to overcome their love for the advancement of the profession.—Ed.]

